# In Vivo Model of Short-Term Efficacy and Favorable Safety of Botulinum Toxin Type E Compared with Type A

**DOI:** 10.3390/toxins18050231

**Published:** 2026-05-16

**Authors:** Jeong-Sun Nam, Daewon Yoon, Yujin Kim, Su-Young Kim, Jae-Young Kim, Yoonkyoung Cha, Joon Seok, Beom Joon Kim

**Affiliations:** 1 Department of Dermatology, College of Medicine, Chung-Ang University, Seoul 06974, Republic of Korea; jsnam@jetema.com (J.-S.N.); yoondw0221@cau.ac.kr (D.Y.); yujinnie4@gmail.com (Y.K.); sykim940714@naver.com (S.-Y.K.); 2 Department of Medicine, Graduate School, Chung-Ang University, Seoul 06973, Republic of Korea; 3 JETEMA, Global 225-12, Pangyoyeok-ro, Bundang-gu, Seongnam 13494, Republic of Korea; jykim@jetema.com (J.-Y.K.); yoonkyoung@jetema.com (Y.C.)

**Keywords:** botulinum toxin, botulinum toxin type A, botulinum toxin type E, muscle atrophy

## Abstract

Botulinum toxin suppresses neurotransmitter release, thereby inhibiting muscle contraction and inducing flaccid paralysis. Botulinum toxin type A (BoNT/A) is widely used for neuromuscular blockade but, upon repeated administration, may cause long-lasting muscle atrophy, fibrosis, and inflammation. It is produced as a single peptide chain that becomes activated through cleavage into a heavy and light chain. BoNT/E, like BoNT/A, is produced as a single-chain polypeptide and requires cleavage to generate the active dichain form. Although BoNT/E is known to have a faster onset and shorter duration of action compared with BoNT/A, its efficacy and safety have not been thoroughly investigated. We compared BoNT/E and BoNT/A in SKH-1 hairless mice. Neuromuscular blockade, recovery pattern, and changes in muscle weight, volume, fiber size, fibrosis, mast cell infiltration, and diffusion to adjacent muscles were evaluated over time. BoNT/E induced maximal neuromuscular blockade on day 3 and fully recovered by day 35, whereas BoNT/A reached maximal effect on day 7 and showed only 20% recovery of the vehicle group by day 35. BoNT/E caused transient, dose-dependent reductions in muscle weight, volume, fiber size, and fibrosis, which largely normalized by day 35. In contrast, BoNT/A, administered at a dose of 0.5 U per injection site, induced persistent muscle atrophy, fibrosis, and significantly increased mast cell infiltration under the experimental conditions used in this study. Neither BoNT/E nor BoNT/A showed diffusion to adjacent muscles or changes in body weight. These findings suggest that BoNT/E provides rapid onset, short duration, and favorable safety, supporting its potential as an alternative therapeutic option for indications requiring temporary muscle relaxation with minimized long-term adverse effects.

## 1. Introduction

Botulinum toxin is one of the most toxic biological substances, which is a neurotoxin produced by the Gram-positive anaerobic bacterium Clostridium botulinum [1]. It is classified into eight serotypes: A, B, C [C1, C2], D, E, F, G, and H, and only toxins A, B, E, and F cause botulism in humans [2]. Especially among the many serotypes, type A is mainly used in both cosmetic and therapeutic applications. Botulinum toxin is composed of a heavy chain (100 kDa) and a light chain (50 kDa), which together form the functional neurotoxin, forming a complex surrounded by hemagglutinin (HA) and nontoxic nonhemagglutinin protein (NTNH) [3,4]. The heavy chain consists of a 50 kDa C-terminal domain (HC) that specifically binds to receptors on cholinergic neurons and a 50 kDa *N*-terminal domain (HN) that induces membrane translocation. The light chain is a zinc-endopeptidase that degrades vesicle-associated membrane protein (VAMP), synaptosome-associated protein (SNAP), and syntaxin, which are involved in the release of the neurotransmitter acetylcholine from synaptic terminals to muscle nerves, thereby triggering the blockade of neurotransmitter release. Depending on the toxin type, the type and site of the target peptide function differently [5,6].

Muscle contraction occurs through signal transmission by neurotransmitters. Nerve cells release a neurotransmitter called acetylcholine, and muscle cells expressing acetylcholine receptors receive the signal and follow the contraction command. After botulinum toxin enters the neuronal cytoplasm, it degrades the soluble *N*-ethylmaleimide-sensitive factor attachment protein receptor (SNARE) protein involved in the release of neurotransmitters. This inhibits acetylcholine release and muscle contraction, inducing flaccid paralysis symptoms [7]. Botulinum toxin is a substance with a high medical value as it is used not only for cosmetic purposes but also for the treatment of cerebral palsy [8,9], dystonia [10,11], urinary incontinence [12,13], migraine [14], and depression [15,16]. Botulinum toxin was first used to treat blepharospasm caused by muscle tremors [17], and the application points of botulinum toxin are very diverse, including the cheeks, glabella, eye area, and chin. If the desired goal and the dosage (unit) of botulinum toxin used do not match, not only will the duration of effect vary, but there is also a risk of side effects [18]. In particular, the blockade of neurotransmitters due to botulinum toxin administration causes muscle atrophy and reduced muscle fiber size [19]. This muscle atrophy, if sustained for a long period of time, can lead to a decline in muscle function. Furthermore, repeated cycles of muscle fiber damage and recovery induce muscle fibrosis, a process that leads to the proliferation of hard fibrous tissue within the muscle, resulting in collagen deposition within the muscle, which impairs muscle elasticity and contractile function [20]. Moreover, botulinum toxin injection sites may be accompanied by an inflammatory response, and mast cell activation, in particular, is known to be involved in the local inflammatory response and tissue remodeling process [21,22]. These changes are closely related not only to the efficacy of botulinum toxin but also to its safety, and therefore can serve as important evidence for evaluating its clinical applicability.

Botulinum toxin type E (BoNT/E) has the same mechanism of action as botulinum toxin type A (BoNT/A) but differs in the cleavage site of SNAP-25. Additionally, BoNT/E requires a shorter time to take effect than BoNT/A. BoNT/A lasts about 26 weeks at its highest concentration, while BoNT/E lasts about 7 weeks [23]. According to a clinical study by Yoelin, S.G. et al., the used BoNT/E began to take effect within 24 h, and the effect lasted from day 14 to day 30 [24]. BoNT/A is produced as a single peptide chain with a molecular weight of about 150 kDa. This chain is initially inactive but becomes active through cleavage into a heavy chain and a light chain. Activated BoNT/A acts as an enzyme that degrades SNARE proteins, thereby blocking the release of synaptic transmitters at the neuromuscular junction. Similarly, BoNT/E also requires proteolytic cleavage by host proteases to become fully active. However, BoNT/E differs from BoNT/A in its activation kinetics and toxin complex composition. In addition, BoNT/E exists only as a 12S (300 kDa) progenitor toxin, whereas BoNT/A exists in all three forms: 12S (300 kDa), 16S (500 kDa), and 19S (900 kDa). This means that BoNT/A can exist in complex forms of various sizes [25].

While BoNT/A is extensively used in esthetic dermatology, BoNT/E has historically received limited attention due to its short duration of action [26,27]. However, recent clinical investigations have begun to re-evaluate this characteristic as a potential therapeutic advantage rather than a limitation. A recent study reported that BoNT/E reduced itching and pain during the acute phase of wound healing, suggesting that a shorter duration of effect may be sufficient or even preferable for certain clinical indications [28]. Despite this emerging interest, in vivo comparisons assessing the detailed efficacy and safety profiles such as muscle atrophy and fibrosis of BoNT/E remain exceedingly scarce. Therefore, we hypothesized that the unique characteristics of BoNT/E might offer a distinct and favorable safety profile, positioning it as a novel alternative for both esthetic and therapeutic applications in dermatology. BoNT/A was selected as a comparator, as it represents the most widely used and clinically established botulinum toxin in both therapeutic and esthetic indications, providing a clinically relevant reference for evaluating the biological characteristics of BoNT/E. In this study, we aimed to compare the efficacy and safety of BoNT/A and BoNT/E by injecting them into mice.

## 2. Results

### 2.1. Muscle-Blocking Effect of BoNT/E Compared to BoNT/A

**Figure 1:**
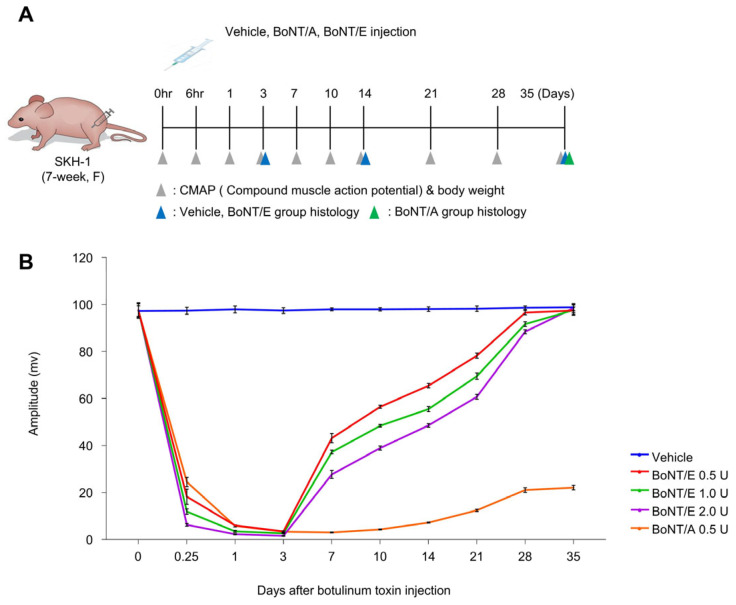
The neuroblocking effect of botulinum toxin. (**A**) Experiment scheme. (**B**) Changes in Compound Muscle Action Potentials (CMAPs) following botulinum toxin injection. *n* = 8/group. Data are the mean ± SD.

CMAP monitoring was performed to determine muscle shrinkage through neurotransmitter blockade by BoNT/E and BoNT/A. BoNT/E showed concentration-dependent blocking effects, with the greatest muscle blocking on day 3. Thereafter, recovery was faster at lower concentrations, reaching a level similar to the vehicle group on day 35. However, BoNT/A showed the greatest muscle blocking on day 7, with recovery reaching 20% of the vehicle group on day 35 (Figure 1B).

### 2.2. Muscle Atrophy Effect of BoNT/E Compared to BoNT/A

To confirm muscle atrophy induced by neurotransmitter blockade due to botulinum toxin, the muscles surrounding the gastrocnemius, which is the injection site, were removed and photographed under a stereomicroscope. The gastrocnemius was then excised, and its weight and volume were measured.

**Figure 2:**
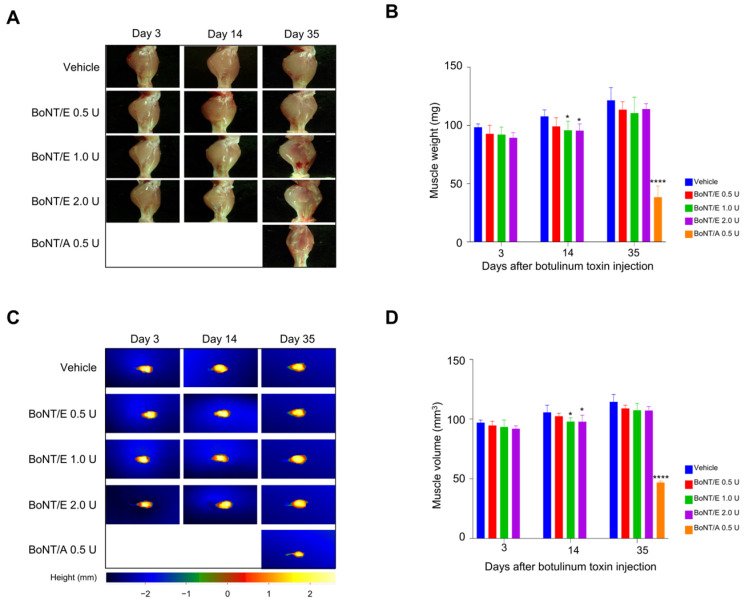
Gastrocnemius weight and volume by botulinum toxin injection. (**A**) Mice right leg picture by DSLR. (**B**) Gastrocnemius weight after botulinum toxin injection. (**C**) Gastrocnemius color-coded height picture by PRIMOS^CR^. (**D**) Gastrocnemius volume. *n* = 5/group. Data are the mean ± SD. Statistical analyses were performed using one-way ANOVA followed by Tukey’s post hoc test. * *p* < 0.05, **** *p* < 0.0001 vs. vehicle.

Macroscopically, the BoNT/E groups showed concentration-dependent leg muscle shrinkage compared to the vehicle group, with similar results on day 35 (Figure 2A). The weight and volume of the injection site, the gastrocnemius, were significantly reduced in the BoNT/E 1.0 U and BoNT/E 2.0 U groups compared to the vehicle group on day 14 but recovered on day 35 to the levels comparable to the vehicle group. However, the BoNT/A group still showed a significant reduction compared to the vehicle group on day 35 (Figure 2B–D).

### 2.3. Muscle Fiber Changes in BoNT/E Compared to BoNT/A

**Figure 3:**
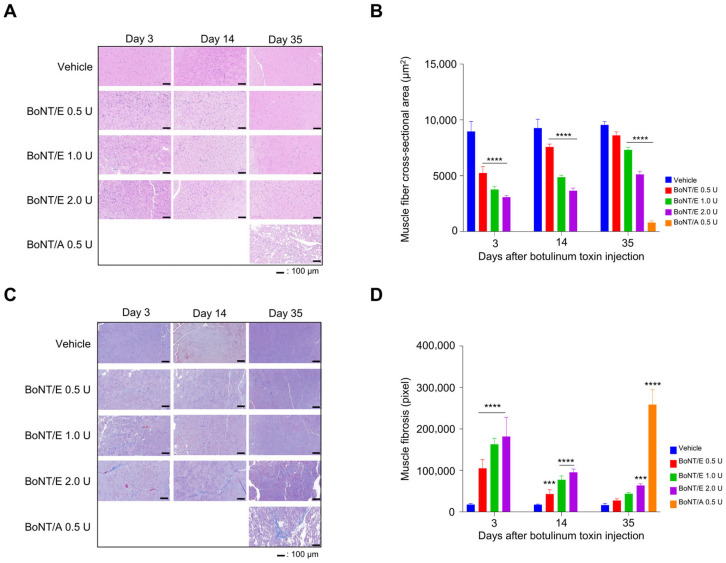
Gastrocnemius fiber cross-sectional area by botulinum toxin injection. (**A**) Representative H&E-stained images of gastrocnemius fiber. (**B**) Gastrocnemius fiber cross-sectional area. (**C**) Representative MT-stained image of gastrocnemius fiber. (**D**) Fibrosis ratio of gastrocnemius fiber. *n* = 5/group. Data are the mean ± SD. Statistical analyses were performed using one-way ANOVA followed by Tukey’s post hoc test. *** *p* < 0.001, **** *p* < 0.0001 vs. vehicle.

The reduction in muscle area induced by botulinum toxin administration was further examined through histopathological analysis. Muscle fiber area decreased in a concentration-dependent manner in the BoNT/E groups compared to the vehicle group on day 3 and recovered in a time-dependent manner. By day 35, the group treated with the lowest concentration (0.5 U) showed muscle fiber area similar to the vehicle group. However, the BoNT/A 0.5 U group still showed a decrease in muscle area compared to the vehicle and BoNT/E 0.5 U groups on day 35 (Figure 3A,B).

Additionally, muscle fibrosis induced by botulinum toxin was confirmed. As evidence, the MT staining area, which represents muscle fibrosis, increased in a concentration-dependent manner in the BoNT/E groups compared to the vehicle group on day 3 (Figure 3C,D). This fibrosis then recovered in a concentration-dependent manner, and by day 35, a significant increase was observed only in the BoNT/E 2.0 U group. However, the muscle fibrosis site increased in the BoNT/A 0.5 U group on day 35 compared to the vehicle group and the BoNT/E 0.5 U group.

### 2.4. Safety of BoNT/E Compared to BoNT/A

**Figure 4:**
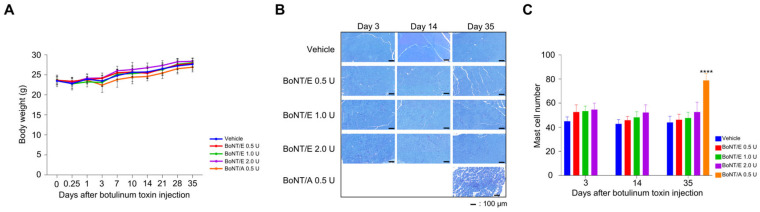
Safety of botulinum toxin. (**A**) Mice body weight after botulinum toxin injection. (**B**) TB staining picture of gastrocnemius fiber. (**C**) Mast cell number. *n* = 5/group. Data are the mean ± SD. Statistical analyses were performed using one-way ANOVA followed by Tukey’s post hoc test. **** *p* < 0.0001 vs. vehicle.

To assess adverse reactions due to botulinum toxin injection, body weights were measured over time after injections of vehicle, BoNT/E, and BoNT/A. The results showed no abnormalities in body weight across all groups of BoNT/E and BoNT/A compared to the vehicle group (Figure 4A). Furthermore, when the number of mast cells was measured to examine inflammatory responses, the BoNT/E group did not show a significant increase in mast cell counts compared to the vehicle group until day 35. However, BoNT/A 0.5 U injection significantly increased mast cell count compared to the vehicle group on day 35 (Figure 4B,C).

### 2.5. Diffusion of BoNT/E Compared to BoNT/A

**Figure 5:**
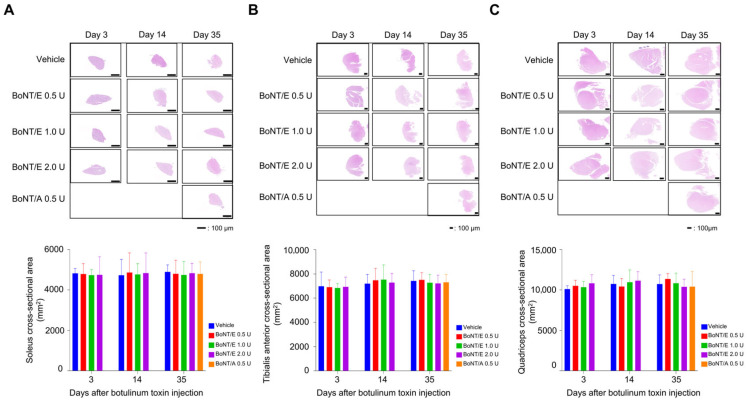
Muscle cross-sectional areas around the botulinum toxin injection site. (**A**) Representative H&E-stained images and cross-sectional area of soleus after botulinum toxin injection. (**B**) Representative H&E-stained images and cross-sectional area of tibialis anterior after botulinum toxin injection. (**C**) Representative H&E-stained images and cross-sectional area of quadriceps after botulinum toxin injection. Scale bar = 100 μm. *n* = 5/group. Data are the mean ± SD. Statistical analyses were performed using one-way ANOVA followed by Tukey’s post hoc test.

To determine whether botulinum toxin affects muscles beyond the injection site, muscle cross-sectional area was measured in three surrounding muscles following injections of vehicle, BoNT/E, and BoNT/A. In all groups of BoNT/E and BoNT/A, the SO, TA, and QF areas remained unchanged compared to the vehicle group, confirming the absence of diffusion (Figure 5A–C).

## 3. Discussion

This study was conducted using the SKH-1 hairless mouse model to compare the physiological and histological effects of BoNT/E and BoNT/A. It is known that botulinum toxin administration blocks the release of the neurotransmitter acetylcholine, causing muscle atrophy, but the atrophied muscles gradually recover as the botulinum toxin effect wears off. In this study, to verify this phenomenon, botulinum toxin was administered to the right lateral gastrocnemius. As a result, BoNT/E showed a maximal muscle-blocking effect on day 3 and then rapidly recovered, reaching almost complete recovery on day 35. In contrast, type A showed a maximal muscle-blocking effect on day 7 and then slowly recovered, reaching only 20% recovery of the vehicle group on day 35, confirming its long-term action.

BoNT/E showed concentration-dependent muscle weight and volume decreases, fiber size atrophy, and fibrosis. However, most of these were recovered by day 35, and the inflammatory response also showed a temporary increase followed by normalization. In contrast, BoNT/A led to sustained muscle atrophy and fibrosis over the same period, with a significant increase in the mast cell count, suggesting a higher potential for an inflammatory response. These findings highlight a significant advantage of BoNT/E regarding muscle quality preservation. Rapid reversibility of BoNT/E prevents the transition from reversible disuse atrophy to irreversible fibrotic degeneration and inflammatory response. Thus, BoNT/E may offer safe esthetic effect by preserving the underlying muscle quality. Overall, BoNT/A exhibited more sustained tissue alterations at later time points, whereas BoNT/E showed faster recovery. However, direct comparisons at early stages are limited due to the absence of time-matched histological data. Although CMAP analysis provided functional insights across all time points, it does not directly reflect tissue-level changes such as muscle atrophy or fibrosis. Therefore, future studies incorporating matched histological assessments at multiple time points for both BoNT/A and BoNT/E will be necessary to enable more comprehensive comparisons of their temporal effects.

BoNT/A has been widely used in clinical settings due to its strong and prolonged neuromuscular blocking effect. It is particularly favored for wrinkle reduction and muscle spasm treatment [29,30]. Furthermore, studies suggest that type A carries a relatively low risk of adverse effects, contributing to its frequent use by physicians [31]. However, as demonstrated in this study, repeated administration of type A may lead to the development of resistance in some patients, highlighting the need for alternative botulinum toxin serotypes. In this regard, BoNT/E appears to be a promising alternative. Its faster onset and shorter duration of action make it particularly useful for conditions requiring short-term muscle relaxation, minimizing long-term side effects. Additionally, BoNT/E showed less tissue damage and a more controlled inflammatory response under the experimental conditions used in this study, suggesting a potentially favorable safety profile compared to BoNT/A. However, confirmation of these findings in clinical settings will require further clinical studies.

Moreover, the unique profile of BoNT/E aligns well with post-surgical scar management where only acute-phase immobilization is required, and rapidly reversible modulation is preferred. And a shorter duration may provide a safer and more flexible option for patients hesitant about long-lasting neuromodulator effects, or for esthetic testing before long-term BoNT/A treatment. The rapid onset and favorable safety profile of BoNT/E make it an ideal candidate for such specific dermatologic procedures.

However, activity units of botulinum toxins are assay-dependent and product-specific and are therefore not directly interchangeable across different serotypes or formulations [32]. Furthermore, even within the same serotype, substantial differences in the amount of neurotoxin protein per unit have been reported among products, indicating that activity units do not necessarily reflect equivalent quantities of active neurotoxin [33]. Accordingly, direct comparison based solely on nominal units may be misleading if interpreted as potency equivalence. Dose-matching based on equivalent biological activity across serotypes was not incorporated into the current study design; as such, normalization may vary depending on the assay system, biological model, and endpoint. Consistent with previous preclinical studies, comparisons between botulinum toxin serotypes are often performed using equi-efficacious dosing approaches rather than nominal unit equivalence [34]. In addition, this study has limitations in that the results were obtained based on an animal model, and atrophy, a well-recognized effect of botulinum toxin, was indirectly examined. Therefore, clinical trials in humans are necessary. Further research is also needed to determine the optimal dose of BoNT/E and whether the effects accumulate with repeated administration. Additionally, efficacy across various indications should be evaluated in future studies, along with mechanistic investigations, such as neuron-based assays assessing SNAP-25 cleavage kinetics and their relationship with functional recovery.

## 4. Conclusions

In conclusion, BoNT/E demonstrated a faster onset, shorter duration of action, and higher safety profile compared to type A. While type A remains widely used due to its strong and prolonged neuromuscular blocking effect, well-established clinical data, and relatively low risk of adverse events, repeated administration may lead to resistance in some patients. Therefore, type E could serve as a valuable alternative for individuals who do not respond to type A or experience side effects. Furthermore, its rapid action and short duration make it a promising candidate for indications requiring fast, temporary muscle relaxation, including acute conditions where minimizing long-term effects is critical.

## 5. Materials and Methods

### 5.1. Materials

Saline (Isotonic Sodium Chloride Inj., Daihan Pharmaceutical Co., Ltd., Seoul, Republic of Korea) served as the vehicle, botulinum toxin type A (BOTOX, Allergan Inc., Irvine, CA, USA) was used for the control group, and botulinum toxin type E (JTM204, JETEMA, Seongnam, Republic of Korea) for the test group. The unit (“U”) used in this study represents a biological activity unit determined using an internal potency assay, with each toxin calibrated against its respective reference standard. Accordingly, activity units are assay-dependent and specific to each toxin preparation and should be interpreted with caution when comparing across different BoNT serotypes. The tested materials were finished drug products, and all dilutions were prepared immediately prior to use under identical handling conditions. Botulinum toxin was reconstituted in saline to obtain the desired concentrations, and 10 μL was injected per site to deliver 0.5–2.0 U per injection.

### 5.2. Mice

For the experiment, 6-week-old SKH-1 hairless mice were purchased from Saeron Bio (Seongnam, Republic of Korea). SKH-1 hairless mice are most commonly used in dermatological research. This mouse strain was selected due to their lack of fur, which facilitates botulinum toxin administration and allows for direct visual observation of inflammation. Additionally, this study was conducted after obtaining approval from the Institutional Animal Care and Use Committee (IACUC) of Chung-Ang University College of Medicine (IACUC: A2022073). All mice were housed in a laboratory maintained under optimal environmental conditions (temperature: 24 ± 2°C; humidity: 50 ± 10%) with a 12 h/day light/dark cycle and were allowed free access to food. A 1-week acclimatization period was provided prior to the start of the experiment.

The mice were randomly divided into five groups: vehicle, botulinum toxin type A (BoNT/A) 0.5 U, and botulinum toxin type E (BoNT/E) 0.5 U, 1.0 U, and 2.0 U. Each test substance (10 μL) was injected into the right lateral gastrocnemius of the mice for the experiment [19]. Body weight and Compound Muscle Action Potentials (CMAPs) were measured in 8 mice per group immediately before injection and 6 h, 1, 3, 7, 10, 14, 21, 28, and 35 days after injection. Histological analysis was performed on 5 mice per point in the vehicle and BoNT/E 0.5 U, 1.0 U, and 2.0 U groups, 3, 14, and 35 days after injection, and in the BoNT/A 0.5 U group 35 days after injection (Figure 1A).

### 5.3. Body Weight

Botulinum toxin was administered to the right lateral gastrocnemius of mice, and changes in body weight were monitored over time to identify any adverse reactions due to botulinum toxin during the study period.

### 5.4. Compound Muscle Action Potentials (CMAPs)

Compound Muscle Action Potential (CMAP) monitoring is measured by using an EMG system (Dantec Keypoint; Natus, Skovlunde, Denmark) to determine the degree of neurotransmitter blockade by botulinum toxin. It detects muscle responses to nerve stimulation as electrophysiological changes within the muscle, thereby measuring functions such as muscle contraction, relaxation, and nerve activity. EMG signals were checked by electrically stimulating the spinal nerves that control leg muscles and measuring the time and magnitude of the response from the active electrode to the reference electrode. The lower the amplitude value of the EMG, the lower the neuromuscular junction activity, indicating a higher ability to block acetylcholine by botulinum toxin administration.

### 5.5. Assessment of Muscle Weight and Volume Change

Botulinum toxin administration blocks neurotransmitters, paralyzing muscles, which results in muscle atrophy and loss of muscle mass. Therefore, the gastrocnemius of mice was imaged under a stereomicroscope (SZ61, Olympus, Tokyo, Japan) after removing surrounding fat and then was isolated and weighed. The isolated gastrocnemius was imaged using a non-contact optical 3D skin imaging device (PRIMOSCR, Canfield Scientific Inc., Parsippany, NJ, USA), and imaging analysis was performed to measure muscle volume. The PRIMOSCR measures volume by refracting parallel transmission fringes with slight height differences on the surface, enabling both qualitative and quantitative analysis. Quantitative assessments were conducted by comparing volumes between groups.

### 5.6. Assessment of Muscle Fiber Cross-Sectional Area Change

For histological analysis, the gastrocnemius was excised and fixed in 10% formalin solution. After preparing a paraffin block, the tissue was cut cross-sectionally to prepare slides, followed by hematoxylin and eosin (H&E) staining. The tissue slides were then scanned at 100× magnification using a slide scanner (PANORAMIC MIDI II, 3DHISTECH Ltd., Budapest, Hungary). In the slide where H&E staining was performed, ImageJ (version 1.52a NIH, Bethesda, MD, USA) was used to measure the area of 10 fibers around the largest muscle fibers adjacent to the injection site in the cross-section of the gastrocnemius, and the changes in muscle fiber area were assessed with the average value.

### 5.7. Muscle Fibrosis Assessment

For histological analysis, the gastrocnemius was excised and fixed in 10% formalin solution. After preparing a paraffin block, the tissue was cut cross-sectionally to prepare slides, followed by Masson’s trichrome (MT) staining. The tissue slides were then scanned at 100× magnification using a slide scanner (PANORAMIC MIDI II, 3DHISTECH Ltd., Budapest, Hungary). In the slide where MT staining was performed, ImageJ (version 1.52a NIH, Bethesda, MD, USA) was used to capture mainly the largest muscle fibers adjacent to the injection site in the cross-section of the gastrocnemius and to measure the percentage of stained collagen fibers to confirm muscle fibrosis.

### 5.8. Confirmation of Inflammatory Response

For histopathological analysis, only gastrocnemius was excised and fixed in 10% formalin solution. After preparing a paraffin block, the tissue was cut cross-sectionally to prepare slides, followed by toluidine blue (TB) staining. The tissue slides were then scanned at 100× magnification using a slide scanner (PANORAMIC MIDI II, 3DHISTECH Ltd., Budapest, Hungary), and the number of mast cells was measured using Case Viewer (version 2.2, 3DHISTECH Ltd., Budapest, Hungary) to assess the degree of inflammation.

### 5.9. Diffusion Assessment

For diffusion assessment, quadriceps femoris, tibialis anterior, and soleus were excised and fixed in 10% formalin solution. After preparing a paraffin block from the fixed tissue, the tissue was cut cross-sectionally to prepare slides, followed by hematoxylin and eosin (H&E) staining. The tissue slides were then scanned at 100× magnification using a microscope (PANORAMIC MIDI II, 3DHISTECH Ltd., Budapest, Hungary). The changes in the cross-sectional area of quadriceps femoris, tibialis anterior, and soleus fibers were measured and evaluated using the Case Viewer program (3DHISTECH Ltd., Budapest, Hungary).

### 5.10. Statistical Significance

All statistical analysis were performed using the statistical program GraphPad Prism 7.0. The experimental results were expressed as mean ± standard deviation (SD). Significance was tested using one-way analysis of variance (ANOVA), and post hoc tests between groups were performed using Tukey’s HSD method, with a *p*-value of 0.05 or less considered statistically significant.

## Data Availability

The original contributions presented in this study are included in the article. Further inquiries can be directed to the corresponding authors.

## Abbreviations

The following abbreviations are used in this manuscript:

BoNT	Botulinum toxin
BoNT/A	Botulinum toxin type A
BoNT/E	Botulinum toxin type E
CMAPs	Compound Muscle Action Potentials
EMG	Electromyography
SNARE	Soluble *N*-ethylmaleimide-sensitive factor attachment protein receptor
VAMP	Vesicle-associated membrane protein
SNAP-25	Synaptosome-associated protein of 25 kDa
NTNH	Nontoxic nonhemagglutinin protein
HA	Hemagglutinin
H&E	Hematoxylin and eosin
MT	Masson’s trichrome
TB	Toluidine blue
SD	Standard deviation
ANOVA	Analysis of variance
QF	Quadriceps femoris
TA	Tibialis anterior
SO	Soleus

## References

[1] Lalli G., Bohnert S., Deinhardt K., Verastegui C., Schiavo G. (2003). The journey of tetanus and botulinum neurotoxins in neurons. Trends Microbiol..

[2] Lebeda F.J., Adler M., Erickson K., Chushak Y. (2008). Onset dynamics of type A botulinum neurotoxin-induced paralysis. J. Pharmacokinet. Pharmacodyn..

[3] Franciosa G., Ferreira J.L., Hatheway C.L. (1994). Detection of type A, B, and E botulism neurotoxin genes in Clostridium botulinum and other Clostridium species by PCR: Evidence of unexpressed type B toxin genes in type A toxigenic organisms. J. Clin. Microbiol..

[4] Hasegawa K., Watanabe T., Suzuki T., Yamano A., Oikawa T., Sato Y., Kouguchi H., Yoneyama T., Niwa K., Ikeda T. (2007). A novel subunit structure of Clostridium botulinum serotype D toxin complex with three extended arms. J. Biol. Chem..

[5] Sugii S., Sakaguchi G. (1977). Botulogenic properties of vegetables with special reference to the molecular size of the toxin in them. J. Food Saf..

[6] Davies J.R., Liu S.M., Acharya K.R. (2018). Variations in the Botulinum Neurotoxin Binding Domain and the Potential for Novel Therapeutics. Toxins.

[7] Nigam P.K., Nigam A. (2010). Botulinum toxin. Indian. J. Dermatol..

[8] Balaban B., Tok F., Tan A.K., Matthews D.J. (2012). Botulinum toxin a treatment in children with cerebral palsy: Its effects on walking and energy expenditure. Am. J. Phys. Med. Rehabil..

[9] Graham H.K., Aoki K.R., Autti-Ramo I., Boyd R.N., Delgado M.R., Gaebler-Spira D.J., Gormley M.E., Guyer B.M., Heinen F., Holton A.F. (2000). Recommendations for the use of botulinum toxin type A in the management of cerebral palsy. Gait Posture.

[10] Camargo C.H., Cattai L., Teive H.A. (2015). Pain Relief in Cervical Dystonia with Botulinum Toxin Treatment. Toxins.

[11] Dressler D., Adib Saberi F., Rosales R.L. (2021). Botulinum toxin therapy of dystonia. J. Neural Transm..

[12] Hsieh P.F., Chiu H.C., Chen K.C., Chang C.H., Chou E.C. (2016). Botulinum toxin A for the Treatment of Overactive Bladder. Toxins.

[13] MacDonald R., Fink H.A., Huckabay C., Monga M., Wilt T.J. (2007). Botulinum toxin for treatment of urinary incontinence due to detrusor overactivity: A systematic review of effectiveness and adverse effects. Spinal Cord..

[14] Schaefer S.M., Gottschalk C.H., Jabbari B. (2015). Treatment of Chronic Migraine with Focus on Botulinum Neurotoxins. Toxins.

[15] Magid M., Reichenberg J.S., Poth P.E., Robertson H.T., LaViolette A.K., Kruger T.H., Wollmer M.A. (2014). Treatment of major depressive disorder using botulinum toxin A: A 24-week randomized, double-blind, placebo-controlled study. J. Clin. Psychiatry.

[16] Kruger T.H., Wollmer M.A. (2015). Depression--An emerging indication for botulinum toxin treatment. Toxicon.

[17] Skopljak-Salkica A., Gabric I., Jagic M., Bejdic N., Biscevic A., Ahmedbegovic-Pjano M. (2024). Clinical Study on the Use of Botulinum Toxin for Blepharospasm and Hemifacial Spasm. Med. Arch..

[18] Bellows S., Jankovic J. (2019). Immunogenicity Associated with Botulinum Toxin Treatment. Toxins.

[19] Na J., Lee E., Kim Y.-J., Choi M.J., Kim S.-Y., Nam J.S., Yun B.J., Kim B.J. (2020). Long-term efficacy and safety of a new botulinum toxin type A preparation in mouse gastrocnemius muscle. Toxicon.

[20] Dadgar S., Wang Z., Johnston H., Kesari A., Nagaraju K., Chen Y.-W., Hill D.A., Partridge T.A., Giri M., Freishtat R.J. (2014). Asynchronous remodeling is a driver of failed regeneration in Duchenne muscular dystrophy. J. Cell Biol..

[21] Lewitt P.A., Trosch R.M. (1997). Idiosyncratic adverse reactions to intramuscular botulinum toxin type A injection. Mov. Disord..

[22] Coté T.R., Mohan A.K., Polder J.A., Walton M.K., Braun M.M. (2005). Botulinum toxin type A injections: Adverse events reported to the US Food and Drug Administration in therapeutic and cosmetic cases. J. Am. Acad. Dermatol..

[23] Pons L., Vilain C., Volteau M., Picaut P. (2019). Safety and pharmacodynamics of a novel recombinant botulinum toxin E (rBoNT-E): Results of a phase 1 study in healthy male subjects compared with abobotulinumtoxinA (Dysport(R)). J. Neurol. Sci..

[24] Yoelin S.G., Dhawan S.S., Vitarella D., Ahmad W., Hasan F., Abushakra S. (2018). Safety and Efficacy of EB-001, a Novel Type E Botulinum Toxin, in Subjects with Glabellar Frown Lines: Results of a Phase 2, Randomized, Placebo-Controlled, Ascending-Dose Study. Plast. Reconstr. Surg..

[25] Zhang K., Yamamoto Y., Suzuki T., Yokota K., Ma S.B., Fatmawati N.N.D., Oguma K. (2012). Type E Toxins Bind to Caco-2 Cells by a Different Mechanism from That of Type A Toxins. Acta Medica Okayama.

[26] Adler M., Keller J.E., Sheridan R.E., Deshpande S.S. (2001). Persistence of botulinum neurotoxin A demonstrated by sequential administration of serotypes A and E in rat EDL muscle. Toxicon.

[27] Eleopra R., Tugnoli V., Rossetto O., De Grandis D., Montecucco C. (1998). Different time courses of recovery after poisoning with botulinum neurotoxin serotypes A and E in humans. Neurosci. Lett..

[28] Alam M., Vitarella D., Ahmad W., Abushakra S., Mao C., Brin M.F. (2023). Botulinum toxin type E associated with reduced itch and pain during wound healing and acute scar formation following excision and linear repair on the forehead: A randomized controlled trial. J. Am. Acad. Dermatol..

[29] Satriyasa B.K. (2019). Botulinum toxin (Botox) A for reducing the appearance of facial wrinkles: A literature review of clinical use and pharmacological aspect. Clin. Cosmet. Investig. Dermatol..

[30] Carruthers A., Carruthers J. (1998). Clinical indications and injection technique for the cosmetic use of botulinum A exotoxin. Dermatol. Surg..

[31] Witmanowski H., Blochowiak K. (2020). The whole truth about botulinum toxin—A review. Postep. Dermatol. Alergol..

[32] Field M., Splevins A., Picaut P., Van der Schans M., Langenberg J., Noort D., Foster K. (2018). AbobotulinumtoxinA (Dysport^®^), OnabotulinumtoxinA (Botox^®^), and IncobotulinumtoxinA (Xeomin^®^) neurotoxin content and potential implications for duration of response in patients. Toxins.

[33] Frevert J. (2010). Content of botulinum neurotoxin in botox^®^/vistabel^®^, dysport^®^/azzalure^®^, and xeomin^®^/bocouture^®^. Drugs R&D.

[34] Nicholson G.S., Canty D., Southern A., Whelan K., Brideau-Andersen A.D., Broide R.S. (2025). Preclinical evaluation of botulinum toxin type E (TrenibotulinumtoxinE) using the mouse Digit Abduction Score (DAS) assay. Toxins.

